# Long Non-coding RNAs: Regulators of the Activity of Myeloid-Derived Suppressor Cells

**DOI:** 10.3389/fimmu.2019.01734

**Published:** 2019-07-25

**Authors:** Gabriela Leija Montoya, Javier González Ramírez, Jorge Sandoval Basilio, Idanya Serafín Higuera, Mario Isiordia Espinoza, Rogelio González González, Nicolás Serafín Higuera

**Affiliations:** ^1^Facultad de Medicina, Universidad Autónoma de Baja California, Mexicali, Mexico; ^2^Facultad de Enfermería, Universidad Autónoma de Baja California, Mexicali, Mexico; ^3^Laboratorio de Biología Molecular, Universidad Hipócrates, Acapulco de Juárez, Mexico; ^4^Facultad de Medicina, Universidad Autónoma de Baja California, Tijuana, Mexico; ^5^División de Ciencias Biomédicas, Departamento de Clínicas, Centro Universitario de los Altos, Universidad de Guadalajara, Tepatitlán de Morelos, Guadalajara, Mexico; ^6^Departamento de Investigación, Universidad Juárez del Estado de Durango, Durango, Mexico; ^7^Unidad de Ciencias de la Salud, Facultad de Odontología, Universidad Autónoma de Baja California, Mexicali, Mexico

**Keywords:** myeloid-derived suppressor cell, long non-coding RNA, inflammation, cancer, immunosuppressive function, accumulation

## Abstract

Myeloid-derived suppressor cells (MDSCs) are a heterogeneous cell population with potent immunosuppressive functions. They play major roles in cancer and many of the pathologic conditions associated with inflammation. Long non-coding RNAs (lncRNAs) are untranslated functional RNA molecules. The lncRNAs are involved in the control of a wide variety of cellular processes and are dysregulated in different diseases. They can participate in the modulation of immune function and activity of inflammatory cells, including MDSCs. This mini review focuses on the emerging role of lncRNAs in MDSC activity. We summarize how lncRNAs modulate the generation, recruitment, and immunosuppressive functions of MDSCs and the underlying mechanisms.

## Introduction

The chronic inflammatory conditions typically observed in many diseases can promote the accumulation of myeloid-derived suppressor cells (MDSCs) (1). This heterogeneous cell population with a strong immunosuppressive function has been principally studied in cancer. However, in recent years, the role of MDSCs has been assessed in other conditions, such as diabetes mellitus, obesity, autoimmune diseases, and infectious diseases (2–4).

Different subsets of MDSCs have been reported; in mice, monocyte MDSCs (M-MDSCs) are described as cluster of differentiation (CD)11b^+^Ly6G^−^Ly6C^+^ cells, and polymorphonuclear MDSCs (PMN-MDSCs) as CD11b^+^Ly6G^+^Ly6C^low^. In humans, M-MDSCs are characterized as CD11b^+^CD33^+^HLADR^−^CD14^+^, whereas PMN-MDSCs are defined as CD11b^+^CD33^+^HLADR^−^CD15^+^ (5). However, other phenotypes have been described in different tumors and infectious diseases (2, 4, 6).

In addition to their high heterogeneity, MDSCs present functional heterogeneity (2). The immunoregulatory functions of MDSCs include the generation of immunosuppressive cells (e.g., regulatory T cells and M2 macrophages) by the production of interleukin (IL)-10; the production of reactive oxygen species (ROS) using the isoforms of nicotinamide adenine dinucleotide phosphate (NADPH) oxidase (NOX1, NOX2, NOX3, and NOX4); and production of reactive nitrogen species, predominantly nitric oxide (NO), by the activation of inducible nitric oxide synthase (iNOS). These reactive species can inhibit the proliferation of T cells, induce the apoptosis of T cells, and reduce both the expression of the ζ chain of T-cell receptors (TCRs), as well as TCR nitration. Moreover, NO can induce the expression of cyclooxygenase 2 (COX2), which regulates the production of prostaglandin-E2, an important molecule that promotes the upregulation of IL-10 and arginase-1 (Arg-1) expression (1). In addition, MDSCs impair the metabolic functions and proliferation of T cells by producing Arg-1, NOS, arginine-glycine amidinotransferase, and L-arginine decarboxylase. The MDSCs can express regulatory molecules, such as programmed death-ligand-1 and Fas ligand to induce the anergy and apoptosis of T cells (1).

The MDSCs originate from common myeloid progenitors in the bone marrow. In addition, extramedullary myelopoiesis in pathologic conditions can generate MDSCs (7, 8). The chronic inflammatory stimuli generated in cancer or infections can induce “emergency myelopoiesis,” which is characterized by the expansion of immature myeloid cells to counterbalance the loss of cells (9, 10). Previously, a “two-signal” model of MDSC accumulation was suggested (11), in which the expansion of immature myeloid cells would be supported by growth factors, such as granulocyte-macrophage colony-stimulating factor, granulocyte colony-stimulating factor, and macrophage colony-stimulating factor. However, a “first signal” would also be required to maintain these cells in an undifferentiated state. A “second signal” would promote the activation of immunosuppressive functions, and thereby generate MDSCs (11).

Transcription factors, such as signal transducer and activator of transcription (STAT)3, interferon regulatory factor-8, retinoblastoma protein (RB)1, and CCAAT/enhancer binding protein (C/EBP)β have been linked to the first signal. Stimulation of the immunosuppressive program has been related, for example, to the activation of the transcription factor, nuclear factor-kappa B (NF-kB) through myeloid differentiation factor (MyD)88, the activation of the STAT1 and STAT6 pathways, and endoplasmic reticulum stress-related pathways associated with the transcription factor, C/EBP homologous protein (CHOP) (11, 12). In addition, the factors associated with the two signals could overlap (11, 12). For example, C/EBPβ, a member of the C/EBP leucine zipper domain-containing family, has three isoforms because of different initiation codons: the liver-inhibitory protein (LIP), liver-activating protein (LAP), and full-length liver-activating protein (LAP^*^). The LAP and LAP^*^ have been considered transcription activators, whereas LIP is considered a repressor or a dominant negative inhibitor of other C/EBP family members (13, 14). Furthermore, C/EBPβ is involved in the regulation of “emergency granulopoiesis” generated by infections or cytokines (15). McPeak et al. showed that reduced expression of C/EBPβ in the myeloid cells of conditional knockout mice was associated with reduced accumulation of MDSCs in a polymicrobial sepsis model (16). In addition, when all CEBPβ isoforms were deleted in hematopoietic lineage cells using tumor mouse models, MDSC accumulation was diminished. Moreover, the MDSCs of these mice showed reduced activity and production of Arg-1 and iNOS proteins (14). *In vitro* studies have suggested that the LAP isoform can bind specific sequences in the regulatory regions of *Arg-1, COX2, NOX2*, and *iNOS*, and promote their expression in MDSCs. In addition, LIP can supposedly interact with LAP to inhibit its transcription function (17, 18). Thus, C/EBPβ could regulate the expansion and suppression of MDSCs.

The MDSCs have been considered to be central regulators in tumor microenvironments. Elimination of MDSCs by targeting the pathways or molecules involved in their generation, expansion, activation, or recruitment at distant sites, and immunosuppressive function in the local microenvironment could improve the response to cancer treatment (19). Thus, a deeper understanding of the mechanisms involved in the control of these processes is important.

In this context, the epigenetic regulation of the biologic behavior of MDSCs has emerged as a novel field and promising tool in therapy (20). “Epigenetics” refers to heritable changes without variations in DNA sequences, and the study of chromatin (21). Thus, epigenetic mechanisms analyzed in MDSCs involve DNA methylation, histone modifications, and regulation by non-coding RNAs (2, 20, 22). In this review, we summarize current knowledge about the central role of long non-coding RNAs (lncRNAs), a type of non-coding RNA that modulates the generation, recruitment, and immunosuppressive function of MDSCs (Table 1). In addition, the underlying molecular mechanisms will be described in some cases.

**Table 1:** LncRNAs and their mechanisms implicated in the accumulation and function of myeloid-derived suppressor cells (MDSCs).

**Name**	**Target/mechanism**	**Biologic effects on MDSCs**	**Associated disorders**	**References**
lnc-C/EBPβ	lnc-C/EBPβ binding to the LIP isoform promotes interaction with the LAP isoform and stops its activity	lnc-C/EBPβ impedes the immunosuppressive function of MDSCs	Patients with rectal cancer or colon cancer overexpress lnc-C/EBPβ in MDSCs Inflammatory and tumor environments stimulate expression of lnc-C/EBPβ in MDSCs *in vitro* and *in vivo*	(18)
lnc-CHOP	lnc-CHOP binds to the LIP isoform and CHOP, and contributes to LAP activation lnc-CHOP instigates accumulation of the epigenetic marker H3K4me3, commonly associated with transcription activation, in the promoters of Arg-1, NOX2, iNOS, and COX2	lnc-CHOP promotes the immunosuppressive activity and generation of MDSCs	Inflammatory and tumor environments stimulate expression of lnc-CHOP in MDSCs *in vitro* and *in vivo*	(17)
RNCR3	RNCR3 sponges mir-185-5p to increase CHOP expression	RNCR3 promotes the generation and immunosuppressive capacity of MDSCs	Inflammatory and tumor environments stimulate expression of RNCR3 in MDSCs *in vitro* and *in vivo*	(23)
Olfr29-ps1	Olfr29-ps1 sponges miR-214-3p and promotes MyD88 expression N6-methyladenosine modification in Olfr29-ps1 is necessary to exert these effects	Olfr29-ps1 promotes the accumulation and immunosuppressive activity of MDSCs	Increased expression of Olfr29-ps1 is observed in the MDSCs of patients with colon cancer or rectal cancer Inflammatory and tumor environments stimulate expression of Olfr29-ps1 in MDSCs *in vitro* and *in vivo*	(24)
Pvt1	Not described	Pvt1 promotes the immunosuppressive activity of MDSCs	Hypoxic, inflammatory and tumor environments stimulate expression of Pvt1 in MDSCs *in vitro* and *in vivo*	(25)
MALAT1	Not described	MALAT1 negatively regulates MDSC generation	Low expression of MALAT1 has been reported in the PBMCs of patients with lung cancer showing increased proportions of MDSCs	(26)
HOTAIRM1	HOTAIRM1 increases expression of HOXA1, which reduces Arg-1 levels and ROS production in MDSCs	HOTAIRM1 promotes MDSC generation	Decreased expression of HOTAIRM1/HOXA1 has been observed in the MDSCs of patients with lung cancer Negative association between the expression of HOTAIRM1/HOXA1 and presence of MDSCs, as well as Arg1 levels has been observed in patients with lung cancer Positive association between expression of HOTAIRM1/HOXA1 and proportions of Th1/cytotoxic T cells in patients with lung cancer	(27)
RUNXOR	RUNXOR reduces RUNX1 expression	RUNXOR promotes the generation and suppressive activity of MDSCs	Increased expression of RUNXOR and decreased expression of RUNX1 in the MDSCs of patients with lung cancer Positive association between expression of RUNXOR and presence of MDSCs, as well as Arg-1 levels in patients with lung cancer; in contrast, a negative association has been observed with RUNX1 Negative association between RUNXOR expression and proportions of Th1/cytotoxic T cells in patients with lung cancer	(28)
HOTAIR	Not described	Increased expression of HOTAIR in hepatocellular carcinoma cell lines promotes MDSC generation	Negative association between HOTAIR expression and the presence of MDSCs in the blood of HPV-positive patients with HNSCC	(29, 30)
PROM1 CCAT1 MUC19	Not described	Not determined	Negative association between expression of PROM1, CCAT1, and MUC19, and the presence of MDSCs in the blood of patients with HPV-positive HNSCC	(29)

## lncRNAs

The lncRNAs are transcripts larger than 200 nucleotides without coding capacity (31). It has been predicted that the human genome encodes >28,000 lncRNAs, most of which are uncharacterized (32). The biogenesis of lncRNAs has several similarities with the biogenesis of messenger RNA (mRNA) (33). In most cases, lncRNA production is carried out by RNA polymerase II. Modifications include the elimination of introns and the addition of a poly-A tail at the 3′ end, and many (but not all) lncRNAs have a cap in their structure at the 5′ end (33–35).

Based on their genomic localization, lncRNAs can be classified as “intronic” (encoded in the introns of genes), “intergenic” (encoded in the regions between two genes), “enhancer” (encoded in the regions of enhancer promoters), “bidirectional” (encoded in the vicinity of a gene of the opposite strand), “sense-overlapping” (encoded in the introns and exons of different genes in the sense strand of DNA), and “antisense” (encoded in the antisense strands of DNA) (32).

The lncRNAs are highly heterogeneous and have substantial functional versatility based on their capacity to be adapted to different structures and molecular interactions (36). In the nucleus, lncRNAs can act as regulators of transcription (regulating DNA methylation, joining transcription factors, and modifying chromatin); be involved in RNA processing (by means of splicing and antisense alignment); act as “molecular decoys” for proteins, such as p53; and be precursors of microRNAs (miRNAs). In the cytoplasm, they can act as molecular decoys for miRNAs, and “scaffolds” for cytoplasmic proteins (37).

Although only a small proportion of all identified lncRNAs have been studied in depth, they are fundamental in many cellular contexts and diverse biological processes (38). In this context, these functional transcripts regulate the differentiation of megakaryocytes, granulocytes, monocytes, and macrophages, and modulate the inflammatory response (39, 40).

## Expression and Function of lncRNAs in MDSCs

### lnc-C/EBP**β**

The lnc-C/EBPβ (also named E130102H24Rik) is an intergenic lncRNA conserved in mice and humans that is encoded in chromosome 1 and chromosome 4, respectively. In addition, it has been found predominantly in cellular nuclei. High expression of lnc-C/EBPβ in the myeloid cells (e.g., macrophages and dendritic cells) of mice has been reported. Importantly, differential expression of lnc-C/EBPβ has been observed in mouse MDSCs if variations in the inflammatory environment occur. Moreover, IL-6 can promote lnc-C/EBPβ expression in MDSCs. Expression of lnc-C/EBPβ has also been reported in M-MDSCs, as well as the PMN-MDSCs of patients with colon cancer or rectal cancer (18). The lnc-C/EBPβ can inhibit expression of enzymes, such as Arg-1, iNOS, NOX2, and COX2, in mouse MDSCs and human MDSC-like cells, resulting in lower concentrations of their metabolic products.

Overexpression of lnc-C/EBPβ in MDSCs promotes the expression of interferon (IFN)-γ in T cells. Tumors in murine models show slower growth if mice are treated with MDSCs overexpressing lnc-C/EBPβ, and tumor-infiltrating T cells demonstrate increased expression of IFN-γ, as compared with controls (18). Thus, lnc-C/EBPβ can attenuate the immunosuppressive function of MDSCs. The suggested mechanism by which this is achieved is the binding of lnc-C/EBPβ to C/EBPβ (specifically to the LIP isoform), which promotes the interaction of LIP with the transcription activator LAP. These interactions prevent the accumulation of LAP in the promoters of *Arg-1, iNOS, NOX2*, and *COX2*, resulting in decreased expression of these enzymes (18).

Expression of lnc-C/EBPβ can block the generation of murine M-MDSCs (18). Because lnc-C/EBPβ has conserved expression and negatively regulates the differentiation and immunosuppressive activity of MDSCs, it could be a potential target for future studies in immunotherapy. To our knowledge, only one report has analyzed this lncRNA. Hence, further studies on the additional functions of lnc-C/EBPβ in various physiological processes and cancer, as well as the molecular mechanisms involved in its regulation of the immune response are necessary.

### lnc-CHOP

The lnc-CHOP (also named GM16727) is an intronic lncRNA that has not been widely characterized. It is encoded in chromosome 11 and localized in cellular nuclei. Interleukins, such as IL-6 and tumor necrosis factor (TNF)-α, and tumor-associated factors can induce the increased expression of lnc-CHOP in mouse MDSCs. Overexpression of lnc-CHOP promotes the expression of Arg-1, NOX2, iNOS, and COX2 and their metabolites in MDSCs, and contributes to the reduction of IFN-γ produced by T cells (17). Thus, lnc-CHOP fosters the immunosuppressive activity of mouse MDSCs.

Furthermore, lnc-CHOP positively regulates MDSC generation and promotes tumor growth in murine models. It has been suggested that lnc-CHOP binds to the transcription factor CHOP and the C/EBPβ isoform, LIP. This allows the activation of LAP and its accumulation in the promoters of target genes, thereby promoting the expression of Arg-1, NOX2, NOS2, and COX2. In addition, increased expression of these enzymes could be the result of enrichment of trimethylation of the amino acid, lysine, at position 4 in the histone H3 (H3K4me3) marker of their promoter regions. The H3K4me3 marker is usually enriched at active chromatin regions and its accumulation is promoted by overexpression of lnc-CHOP in MDSCs (17). The lnc-CHOP could use different mechanisms to promote the accumulation and activation of MDSCs. The conservation and role of lnc-CHOP in human MDSCs, as well as its contribution in tumor biology, has yet to be determined. Future studies could ascertain the additional functions of lnc-CHOP and its potential applications.

### Retinal Non-coding RNA (RNCR)3

The RNCR3 is an intergenic lncRNA that is highly conserved in mammals (in which it is also known as LINC00599) (41). The RNCR3 expression is reportedly related to glioblastoma, prostate cancer, atherosclerosis, and retinal microvascular abnormalities (41–44). Mouse MDSCs express nuclear and cytoplasmic RNCR3, the expression of which is increased in mice with tumors. In addition, IL-6 induces the increased expression of this lncRNA in the MDSCs of mice (23). Downregulation of RNCR3 expression prevents MDSC differentiation *in vitro* and *in vivo*, whereas RNCR3 expression promotes the preferential differentiation of PMN-MDSCs. Importantly, RNCR3 contributes to the immunosuppressive function of MDSCs to induce the expression of Arg-1 and iNOS *in vitro*.

Furthermore, IFN-γ production by T cells is increased in the presence of MDSCs with reduced expression of RNCR3. A tumor model in mice treated with MDSCs down-regulating the expression of RNCR3 showed increased tumoral growth (23). One possible mechanism for this is the use of RNCR3 to “sponge” mir-185-5p. The latter impedes MDSC generation and the production of INOS and Arg-1 by targeting CHOP. In the presence of RNCR3, mir-185-5p binds to it preferentially, resulting in the upregulation of CHOP expression (23). The immunosuppressive function of MDSCs is promoted by CHOP, as CHOP-deficient MDSCs show increased expression of the LIP isoform and reduced binding of C/EBPβ to promoters of Arg-1 and IL-6. This results in the reduced expression of IL-6 and activation of STAT3, as well as impaired immunosuppressive function (45). Thus, RNCR3 supports the accumulation and immunosuppressive program of MDSCs. Additional studies could determine whether RNCR3 exerts biological effects on human MDSCs for potential applications against chronic inflammatory diseases in humans.

### Olfactory Receptor 29, Pseudogene 1 (Olfr29-ps1)

The Olfr29-ps1 is a lncRNA in mice (OR1F2P in humans) that has not been characterized previously. It is conserved and expressed in the nuclei and cytoplasm of murine and human MDSCs and macrophages. Tumor-associated factors and IL-6 can increase the expression of Olfr29-ps1 in the MDSCs of mice. Mononuclear cells with a MDSC phenotype in patients with colon cancer or rectal cancer have shown increased expression of Olfr29-ps1 (24). The overexpression of Olfr29-ps1 in bone marrow cells (BMCs) has been shown to promote the generation of mouse M-MDSCs in a differentiation model using cellular cultures, and an *in vivo* BMC chimera model. In addition, augmented accumulation of M-MDSCs has been observed in the tumors and spleen of a tumor model in mice with Olfr29-ps1-overexpressing MDSCs. The immunosuppressive activity of human and murine MDSCs is increased by Olfr29-ps1 overexpression (24).

*In vitro* analysis showed that IFN-γ production by T cells is reduced in the presence of Olfr29-ps1-overexpressing MDSCs, and that these MDSCs show increased protein expression of Arg-1, COX2, NOX2, and iNOS, as well as increased production of their metabolites. Tumors in a mouse model with Olfr29-ps1-overexpressing MDSCs show greater growth and fewer infiltrating T (especially CD^8+^) cells. *In vitro* analyses suggest that the effects generated by Olfr29-ps1 could be explained (at least in part) by its capacity to sponge miR-214-3p (24). The latter inhibits the expression of the mRNA and protein expression of MyD88, so interactions between Olfr29-ps1 and miR-214-3p result in the augmented expression of MyD88 (24). Thus, the immunosuppressive activity of MDSCs is promoted by MyD88.

Interestingly, modification of N6-methyladenosine in regions of the Olfr29-ps1 sequence is essential for the stability and function of Olfr29-ps1 in MDSCs. This modification is common in mRNAs and generated by methyltransferases, such as methyltransferase-like (METTL)3. Notably, the downregulation of METTL3 expression reduces Olfr29-ps1 production, as well as the immunosuppressive activity and generation of MDSCs (24). Mechanistically, these observations are important because modification of N6-methyladenosine is reversible and could have potential therapeutic benefits. However, only additional research will show whether this is possible.

### Plasmacytoma Variant Translocation (Pvt)1

The Pvt1 is an intergenic lncRNA. It is conserved in humans and mice. Notably, it is over-expressed in several human cancers, including melanoma, cervical cancer, gastric cancer, prostate cancer, hepatocellular cancer, esophageal cancer, and acute myeloid leukemia (25, 46, 47). Tumor-infiltrating PMN-MDSCs and M-MDSCs show increased expression of Pvt1 in tumor mouse models, and overexpression in the splenic MDSCs of those mice. In addition, the presence of IL-6 increases the expression of Pvt1 in PMN-MDSCs generated in cultures. Interestingly, hypoxic conditions and expression of hypoxia-inducible factor (HIF)-1α increases Pvt1 production in PMN-MDSCs *in vitro* (25). Thus, inflammatory and tumor microenvironments could promote the increased expression of this lncRNA in MDSCs.

Downregulation of Pvt1 expression in PMN-MDSCs can induce reduced production of ROS and Arg-1 activity, as well as a slight increase in T-cell proliferation in co-cultures. A tumor mouse model treated with Pvt1-down-regulating PMN-MDSCs showed reduced growth in the generated tumors and a modest increase in the number of CD8+ T cells producing IFN-γ in lymphatic nodules (25). These results suggest that Pvt1 promotes immunosuppressive activity in PMN-MDSCs. Whether Pvt1 modulates the immunosuppressive functions of human MDSCs warrants future exploration. In addition, the molecular mechanisms involved in MDSC regulation by this lncRNA should be investigated.

Interestingly, similar microenvironmental factors (e.g., IL-6 or tumor-associated factors) can induce the overexpression of lnc-C/EBPβ, lnc-CHOP, Olfr29-ps1, Pvt1, and RNCR3 in MDSCs. These microenvironmental factors produce contrasting effects because lnc-CHOP, Olfr29-ps1, Pvt1, and RNCR3 promote, whereas lnc-C/EBPβ prevents, the immunosuppressive functions and differentiation of MDSCs (17, 18, 23–25). These actions could indicate “fine tuning” of gene regulation and the importance of lncRNAs in the control of the biological behavior of MDSCs. In addition, the final biological effect could result in crosstalk among the diverse pathways regulated by lncRNAs.

### Metastasis-Associated Lung Adenocarcinoma Transcript (MALAT)1

The MALAT1 (also named nuclear-enriched abundant transcript-2) is a nuclear intergenic lncRNA. It is highly conserved among species and involved in various diseases. The MALAT1 is considered an oncogene because it can promote the proliferation, invasion, and metastasis of many types of human cancer cells (48). Thus, this lncRNA has been studied to develop new strategies in the diagnosis and treatment of cancer (48). Using an *in vitro* differentiation model, Zhou and colleagues recently reported that the reduced expression of MALAT1 in peripheral blood mononuclear cells (PBMCs) promotes their differentiation to MDSC-like cells. Interestingly, the reduced expression of MALAT1 has been reported in the PBMCs of patients with lung cancer, as well as an increased proportion of MDSCs (26). Thus, MALAT1 could negatively regulate the differentiation of MDSCs in patients with lung cancer. More studies evaluating the role and mechanisms through which MALAT1 regulates MDSC differentiation in different diseases could lead to new directions in potential therapeutics.

### HOXA Transcript Antisense RNA Myeloid-Specific (HOTAIRM)1

The HOTAIRM1 is an intergenic lncRNA localized between homeobox (*HOX*)*A1* and *HOXA2* genes, and is expressed preferentially in the myeloid lineage. It has been associated with glioblastoma and myeloid leukemia, as well as colorectal, pancreatic, lung, and breast cancer (49, 50). Importantly, HOTAIRM1 regulates the differentiation of myeloid cells (27, 50). Reduced expression of HOTAIRM1 has been reported in the MDSCs of tumors of patients with lung cancer. In addition, the overexpression of HOTAIRM1 has been shown to reduce the differentiation of MDSCs and production of Arg-1 in cellular cultures using human PBMCs (27). Consistent with those data, diminished expression of HOTAIRM1 was found in the PBMCs of patients with lung cancer, as well as increased proportions of MDSCs. Moreover, a negative association was observed between HOTAIRM1 expression and the presence of MDSCs, as well as Arg-1 production; whereas a positive association was observed with respect to the percentage of T-helper (Th)1 cells and cytotoxic T cells in the same patients (27).

The mechanism by which HOTAIRM1 regulates MDSCs could be associated with *HOXA1* expression. The HOTAIRM1 can induce HOXA1 expression in MDSCs, which reduces Arg-1 expression and ROS production. In addition, increased expression of HOXA1 has been shown to reduce tumor growth, decrease the percentage of MDSCs, and enhance the immune response in a tumor mouse model. Moreover, a positive association has been observed between the expression of HOTAIRM1 and HOXA1 in patients with lung cancer (27). These observations suggest that HOTAIRM1 inhibits the differentiation and suppressive activity of human and mouse MDSCs. Further studies analyzing the effects of HOTAIRM1 on MDSCs in other tumor types should be conducted. In addition, the mechanisms involved in the reduced expression of HOTAIRM1 in lung cancer have yet to be determined.

### Runt-Related Transcription Factor-1 Overlapping RNA (RUNXOR)

The RUNXOR is an intragenic lncRNA that has been very rarely studied. It is localized in the locus of the runt-related transcription factor (*RUNX*)*1* gene, and its expression is increased in the bone marrow of patients with acute myeloid leukemia (51). The MDSCs generated in cell cultures using the PBMCs and MDSCs of tissue from patients with lung cancer express high levels of RUNXOR. Furthermore, the down-regulated expression of this lncRNA in PBMCs disturbs their differentiation to MDSCs in a cell-culture model. Moreover, Arg-1 expression is reduced if RUNXOR expression is decreased in MDSCs. Thus, RUNXOR is involved in promoting the generation and immunosuppressive function of MDSCs (28).

Interestingly, the increased expression of RUNXOR has been reported in the PBMCs of patients with lung cancer, and a positive correlation has been described between RUNXOR expression and the presence of MDSCs, as well as Arg-1 production in such patients. In contrast, a negative correlation has been observed between RUNXOR expression and the percentage of both Th1 cells and cytotoxic T cells (28). It has been suggested that RUNXOR exerts its biologic effects on MDSCs through its target RUNX1. Wang and co-workers suggested that RUNXOR binds the enhancer of zeste homolog 2 (histone H3K27 methyltransferase component of polycomb repressive complex 2) and RUNX1 protein to the RUNX1 promoter; and the RUNXOR promoter could compete with the RUNX1 promoter for the transcription machinery (51). Thus, RUNXOR could reduce RUNX1 expression in MDSCs *in vitro* and in patients with lung cancer. Reduced expression of RUNX1 in mouse MDSCs promotes the production of Arg-1, iNOS, and ROS *in vitro*. Moreover, RUNX1 expression induces the differentiation of MDSCs into myeloid cells with a mature phenotype (52). Therefore, RUNXOR could promote the expansion and immunosuppressive activity of MDSCs in lung cancer.

Evidently, MALAT1, HOTAIRM1, and RUNXOR regulate important biological activities (e.g., expansion, differentiation, and immunosuppressive functions) of MDSCs in lung cancer. Hence, these lncRNAs could offer opportunities for potential therapeutic applications against lung cancer; nevertheless, a considerable amount of research would be necessary.

### Hox Antisense Intergenic RNA (HOTAIR)

The HOTAIR is an oncogenic lncRNA positively associated with initiation, growth, angiogenesis, progression, drug resistance, and poor prognosis in cancer (53). The expression of HOTAIR has been indirectly related to MDSC recruitment. A negative association has been reported between HOTAIR expression and the proportion of MDSCs in the blood samples of patients with human papillomavirus-positive head and neck squamous cell carcinoma (HPV-positive HNSCC) (29), but a causal relationship has not been established. Furthermore, HOTAIR overexpression in cell lines of hepatocellular carcinoma can induce increased production of the C-C motif chemokine ligand (CCL)2 (30). The CCL2 is not a specific chemokine for MDSCs and is a chemoattractant for several tumor-related myeloid cells (including monocytes and tumor-associated macrophages). Hence, HOTAIR expression could exert a more generalized function by promoting inflammation and immunosuppression within the tumor microenvironment. In addition, using a differentiation model of PBMCs from human donors, cell lines of hepatocellular carcinoma overexpressing HOTAIR promoted MDSC differentiation in co-cultures (30). Thus, HOTAIR expressed by tumor cells could positively regulate MDSC generation *in vitro*; nevertheless, the associated molecular mechanisms have not been determined. Studies are needed to ascertain the functional role of HOTAIR in the recruitment or differentiation of MDSCs.

### Other lncRNAs

In addition to HOTAIR, three other lncRNAs have been negatively associated with MDSCs in the blood samples of patients with HPV-positive HNSCC: prominin (PROM)1, colon cancer associated transcript (CCAT), and mucin (MUC)19 (29). However, the molecular mechanisms implicated in the recruitment or expansion of MDSCs have not been determined, because a direct molecular role of these lncRNAs in MDSC biology has not been reported. In future studies, these lncRNAs could be evaluated as potential biomarkers in patients with HPV-positive HNSCC, because the proportion of MDSCs is increased in these patients as compared with precancerous lesions and normal oral mucous tissues (29).

To our knowledge, only one study has focused on the relationship between lncRNAs and MDSCs in non-cancer-related diseases. The lncRNA expression was analyzed in the MDSCs generated in mice infected with *Echinococcus granulosus* (54) (the causal agent of cystic echinococcosis in humans). This zoonotic disease principally affects the liver and lungs. In mice infected with *E. granulosus*, the expansion of MDSCs that down-regulate the activity of T cells has been reported (55). These MDSCs, in the presence of this infectious agent, showed 649 differentially expressed lncRNAs. Bioinformatics analyses based on mRNA expression revealed alterations in biologic processes (e.g., signaling by mechanistic target of rapamycin) and the involvement of 288 lncRNAs in the *cis*-regulation of their sense-overlapping genes. Interestingly, *Rb1* regulation by the lncRNA NONMMUT021591s was predicted; 60 transcription factors regulating expression of 372 lncRNAs predicted the regulation of the lncRNA, FR015378, by C/EBPβ (54). Additional studies could determine the biological contribution of lncRNAs in modulating the function and differentiation of MDSCs in the context of infections.

## Conclusions and Future Perspectives

Studies have suggested that specific lncRNAs control the differentiation of MDSC subsets and immunosuppressive function. Furthermore, lncRNAs show specific patterns of expression depending on the cell and tissue types (39). Thus, lncRNAs could be potential specific markers of MDSC subsets and several MDSCs with diverse phenotypes that have been observed in various diseases, but these have yet to be determined. In addition, controversy has been generated because the close relationship among MDSCs, neutrophils, and monocytes. Analyzing the expression profile of lncRNAs in MDSCs compared with that of lncRNAs in myeloid cells could provide new insights into the differences described among these cell types. Studies analyzing lncRNAs exclusively in mouse MDSCs should be viewed with caution, considering that lncRNAs seem to be poorly conserved among various species (56). Therefore, investigations into the modulation of MDSC activity by lncRNAs should consider the evolutionary conservation of lncRNA in human MDSCs for potential applications against human diseases. The lncRNA expression profile in human MDSCs has yet to be reported.

Understanding the regulation of immunosuppressive function and the accumulation of MDSCs to find therapeutic targets that modulate immunosuppression is more important than the classification of MDSCs among myeloid cells. The lncRNAs regulate the activity of different transcription factors (e.g., C/EBPβ, CHOP, and RUNX1) involved in the differentiation and suppression of MDSCs. Thus, these non-coding RNAs might play significant roles in the two-signal model (11), in which lncRNAs (such as Olfr29-ps1, lnc-CHOP, RNCR3, and RUNXOR) could participate in the first phase during expansion of the MDSCs, and then in the second phase to promote MDSC activation. The Pvt1 could participate only in the second phase. The versatility of lncRNAs in the recognition of different targets could facilitate their participation in both phases. In addition, the factors produced by tumors, hypoxia, or an inflammatory microenvironment could support the expression of some non-coding RNAs. Moreover, the chronic and low-dose stimuli generated by inflammatory and tumoral factors could promote the downregulation of lncRNAs that inhibit the accumulation or immunosuppressive function of MDSCs. Future studies will determine whether this perception is correct.

In addition to intracellular regulation, lncRNAs can exert intercellular effects *via* exosomes (57, 58). These extracellular nanovesicles are derived from endosomes, have a diameter of 30–100 nm, and are secreted by different cell types, including cells with a myeloid lineage, such as MDSCs (59, 60). Limited information on MDSC exosomes indicates that they can exert effects associated with immunosuppression and the promotion of tumorigenesis (61). The MDSC exosomes carry proteins, RNAs, and miRNAs (62), but the presence of lncRNAs in MDSC exosomes has not been investigated. Future studies addressing this issue would be important, because lncRNAs are supposedly selectively packaged in exosomes and secreted by cancer cells and stroma cells to modulate the growth, metastasis, angiogenesis, and chemoresistance of cancer cells (57, 58). Moreover, the characterization of lncRNAs in exosomes secreted by myeloid lineage cells is not widely understood.

The lncRNAs involved in the biological behavior of MDSCs could facilitate the development of novel therapeutic approaches. However, if these lncRNAs are involved in multiple physiological functions or have contrasting effects in different cell types, then alteration/manipulation of lncRNAs could also generate undesirable side effects. Thus, one cannot suggest that targeting lncRNAs is feasible or practical. A more comprehensive understanding of lncRNA functions and the molecular mechanisms implicated in the modulation of MDSCs is necessary.

The central role of MDSCs in generating immunosuppressive tumor microenvironments supports the growth and progression of tumor cells. In addition, a general understanding of the modulation of the inflammatory response by MDSCs in other diseases has been improved in recent years. Thus, molecules that regulate the biological behavior of MDSCs could be the targets of therapies against these diseases.

The lncRNAs are involved in the control of MDSC differentiation and immunosuppressive programs in cancer via various molecular mechanisms (Table 1). Nevertheless, the functional link between some lncRNAs and MDSCs does not seem to be sufficiently strong. Thus, the study of the mechanisms by which lncRNAs modulate MDSCs is in its infancy. The lncRNAs control gene expression and diverse biological functions in health and disease in both cell- and tissue-specific manners. Hence, future studies should aim to identify the novel lncRNAs that regulate MDSC activity, so they can be applied in immunomodulatory therapy or as biomarkers.

## Author Contributions

All authors listed have made a substantial, direct and intellectual contribution to the work, and approved it for publication.

### Conflict of Interest Statement

The authors declare that the research was conducted in the absence of any commercial or financial relationships that could be construed as a potential conflict of interest.
